# 

**Published:** 1861-07

**Authors:** 


### CONTENTS.---JULY, 1861.

### ORIGINAL CONTRIBUTIONS.

PAGE

ART. I. Lectures on Military Surgery. By E. ANDREWS, A. M., M. D., Professor of Surgery, etc. Lecture III., ..... 321

ART. II. Death from Prolonged Irritation of the Left Phrenic Nerve. Reported by J. H. HOLLISTER, M. D., ..... 327

ART. III. Cimicifuga Racemosa, SIMPSON, PARRISH and DAVIS, ..... 331

ART. IV. Conservative Surgery. By H. WANZER, M. D., ..... 338

ART. V. A Case of Superfætation—Placenta Præira. By J. H. APPERSON, M. D., ..... 339

### THE CLINIQUE.

MARINE HOSPITAL.—Service, Dr. ISHAM ; reported by A. D. ROUSE, Student of Medicine. Complete Amaurosis, ..... 340
Acute Pleurisy, ..... 341
Syphilitic Rupia, ..... 342
Erythema ; produced by local irritation, ..... 343
MERCY HOSPITAL—Service Dr. N. S. DAVIS. Reported for the EXAMINER. Rubeola,. 344
Typhoid Fever, with Threatened Coxalgia Supervening on Recovery, ..... 350
Epulo-Cancroid Tumor, ..... 352

### SELECTIONS.

Rheumatism. By B. WOODWARD, M. D., Galesburg, Ill., ..... 353

Dr. SIMPSON on Actæa Racemosa, ..... 356

FOREIGN ITEMS : Removal of the Placenta, ..... 357

Transfusion of Blood in Obstetrical Practice, ..... 358

Terebinthinated Caoutchouc in Phthisis, ..... 359

### BOOK NOTICES.

A Practical Treatise on Military Surgery. By FRANK HASTINGS HAMILTON, M. D., ..... 360
Third Annual Report of the Chicago Charitable Eye and Ear Infirmary, ..... 362
The Classification, Diagnosis and Prognosis of Tumors. By Prof. TH. BILLROTH, ..... 364
The Modus Operandi of Various Kinds of Baths, Sea-Bathing, Heat and Cold, Physiologically Explained. By JOHN O'REILLY, M. D., ..... 366
Books Received....London Lancet....The True Physician. By Dr. JOHN WATSON, ..... 367
Transactions of the New Jersey Medical Society, for 1860, ..... 368

### EDITORIAL.

The Influence of Alcoholic Drinks on the Development and Progress of Pulmonary Tuberculosis, ..... 368
Western Army Medical Matters, ..... 372
Army Sanitary Commission, ..... 374
Personal .... Volunteer Women Nurses .... Incompetent Surgeons .... Dr. GIBBS' Summary, ..... 376
Tough Lunar Caustic...A Medical Sharp-Shooter...Absence of the Uterus..."Onward!" 377
Co-existence of Diseases....Conservative Surgery, ..... 378
Chlorate of Potash in Variola, ..... 379
Regeneration of Nerve-Fibre, ..... 380
Cannabis Indica, ..... 381
Special Notices, ..... 383

